# Impact of a First-Year Place-Based Learning Community on STEM Students’ Academic Achievement in their Second, Third, and Fourth Years

**DOI:** 10.1007/s10755-022-09616-7

**Published:** 2022-08-18

**Authors:** Matthew D. Johnson, Steven T. Margell, Katlin Goldenberg, Raven Palomera, Amy. E. Sprowles

**Affiliations:** 1 Department of Wildlife, Humboldt State University, Arcata, CA USA; 2 Sponsored Programs Foundation, Humboldt State University, Arcata, CA USA; 3 Department of Biological Sciences, Humboldt State University, Arcata, CA USA

**Keywords:** Equity gap, Retention, Quasi-experimental design, Graduation, Persistence, GPA

## Abstract

Learning communities for college students have been shown to improve first-year student outcomes and narrow equity gaps, but longer-term data to evaluate whether these benefits persist through multi-year retention and graduation are rare. This is especially important for students in science, technology, engineering and math, who often confront gateway courses and challenging academic cultures in their second and subsequent years. Here, we report on the second, third, and fourth year academic outcomes of three cohorts of a first-year placed-based learning community. Relative to a reference group, participants in the learning community generally showed similar grade acquisition in second- and third-year STEM courses, and initially higher GPAs for learning community participants later diminished to be statistically indistinguishable from the reference group. Nonetheless, units completed after one, two, and three years were slightly higher for learning community participants than for the reference group, and with narrower equity gaps. The learning community also increased and narrowed equity gaps in second- and third-year retention at the institution and in STEM specifically (+6 to +17%). Four-year graduation rates from the institution and in STEM specifically also increased (+8 to +17%), but equity gaps were only narrowed slightly. These results suggest that while benefits of first-year learning communities on grades decline over time, benefits for retention and graduation can persist, though they are insufficient to erase equity gaps. Future work should examine how scaffolding practices in students’ second and third years can better sustain and even magnify inclusive success improvements initiated by first year learning communities.

## Introduction

Innovation derived from advances in science, technology, engineering, and math (STEM) is vital to build a sustainable economy, access renewable energy, maintain public health, avoid environmental collapse, and ensure national security (National Research Council, 2007; Change the Equation, 2015; Maltby et al., 2016; Fayer et al., 2017). However, despite the high demand for STEM occupational talent, low retention and graduation rates in STEM education is a national concern, with less than one-sixth of U.S. high school students pursuing a STEM major and only 50% of entering STEM majors matriculating into STEM fields (US Department of Education, 2015). Moreover, academic cultures typically benefit students from backgrounds similar to those who have historically been successful, and participation in STEM by racially underrepresented students declines at every career step, from K-12 to post-graduate work (Museus et al., 2011; NCES, 2021). Disparities are pronounced in biological and natural resource sciences, with only 20% of Bachelor’s degrees, 17% of Master’s degrees, and 9% of PhDs in these fields awarded to students self-identifying as Hispanic, Black, Pacific Islander, or Native American/Alaskan as of 2019 (NCES, 2020). Degree attainment in STEM also lags for first generation students (first in their family to attend college), and low-income students (Yelamarthi & Mawasha, 2008; Bettencourt et al., 2020), who can especially benefit from the opportunities for economic mobility afforded by STEM degrees. These inequities impact innovation, as evidence from multiple fields now shows that group diversity among STEM teams improves outcomes and novel solutions by bringing in a wider range of voices and perspectives (Hong & Page, 2004; Gibbs, 2014; Jilani, 2021). Therefore, increasing the attainment of STEM degrees, especially by a diverse range of students, is not only essential for improving science, it is a moral imperative (National Academy of Sciences, 2011; Asai, 2020).

First-year learning communities are one of several high impact practices (Kuh, 2008; AAC&U, 2022) widely used by universities to increase the retention and academic success of STEM students, and a large body of evidence suggests they are especially helpful for underrepresented students, first generation students, and low-income students (Graham et al., 2013; Weiss et al., 2015). Learning communities can take many different forms, from interest or thematic groups and first-year experience programs, to programmatic residency-based initiatives (Dean & Dailey, 2020). Here, we refer specifically to learning communities as “a variety of curricular approaches that intentionally link or cluster two or more courses, often around an interdisciplinary theme or problem, and enroll a common cohort of students” (Smith et al., 2009, p. 20). The loss of STEM undergraduates is especially problematic in their first year of college (Clark, 2005), and previous work has shown that learning communities can improve students’ first year outcomes, leading to a rapid increase in their popularity (Smith, 2001; Kuh, 2008). These benefits arise through several mechanisms (Weiss et al., 2015), including strong peer-to-peer relationships when students are co-enrolled into two or more courses (Smith et al., 2009) and improved learning if content is linked across courses (Klein, 2005). Linking STEM content to social and civic issues has been shown to improve learning and engagement (Chamany et al., 2008; Sheardy, 2010; Sadler, 2011; Burns, 2017), particularly for students from groups underrepresented in STEM (Harackiewicz et al., 2016; Estrada et al., 2016). Finally, a greater level of engagement with campus life and the cultivation of academic (STEM) identity and self-efficacy is cultivated in learning communities designed to connect social and student support programs to the curriculum, which can strengthen relationships between faculty, students, and staff (Tinto, 2003). Such psychosocial factors have been shown to be important for first year STEM students and linked to improved outcomes such as first year retention and graduation rates (Carrino & Gerace, 2016; Solanki et al., 2019).

Over nearly twenty years, research has suggested that learning communities have positive effects on student outcomes (Zhao & Kuh, 2004; Graham et al., 2013; Otto et al., 2015; Weiss et al., 2015; Solanki et al., 2019), but few have followed students after their participation in learning communities (Whalen & Shelley, 2010; Dagley et al., 2016). If and how learning communities improve long term academic outcomes remains poorly understood (Cambridge-Williams et al., 2013; Maltby et al., 2016). Nonetheless, the possible long-term benefits of a first year learning community are conceptually grounded by their observed positive impacts on students’ academic skills (Weiss et al., 2015) and psychosocial factors (Solanki et al., 2019), and recent work is beginning to provide some empirical evidence of possible benefits beyond students’ first year. From the few longer-term studies of a STEM learning community, Dagley et al. (2016) showed that a 2-year learning community resulted in, on average, graduation rates that were 12 percentage points higher than a reference group, and Maltby et al. (2016) showed that a first-year living-learning community increased the number of women and underrepresented students attaining an undergraduate degree in STEM. These results are encouraging, and additional studies are needed to establish their generality and to explore more details in students’ academic performance in years after they participated in a learning community, including their pace of completing academic units, GPAs, and pass rates in core STEM courses.

Here, we report on the second, third, and fourth year academic outcomes of students participating in a place-based first-year learning community designed for students in the life sciences and natural resource disciplines. This follows previous work that showed this learning community elevated first year retention rates and grades, particularly for underrepresented, first-generation, and low income students, which helped narrow equity gaps (Johnson et al., 2020). We test the hypothesis that these first-year benefits of the learning community extend into students’ later academic performance, again with particular attention to underrepresented, first-generation, and low income students. We followed the same learning community participants and a reference group of STEM students matched on multiple criteria to be similar to the participants (e.g., high school GPA, race, gender, etc.), and we examined their academic outcomes in their second, third, and fourth years. With this quasi-experimental approach, the primary systematic difference between the treatment group (participants) and reference group was their participation in first-year learning community. Thus, observed differences in long-term academic outcomes between the groups are likely influenced by the learning community participation, though interactive effects of the learning community and subsequent experiences may also be at play (Dagley et al., 2016; Maltby et al., 2016). Specifically, we examined units completed, GPAs, pass rates in core STEM courses, and retention into students’ second, third, and fourth years. Finally, we examined four-year graduation rates both from the institution, and in STEM specifically, as well as students’ self-reported sense of STEM identity and career preparation.

## Program Design and Participants

This study was conducted at a mid-sized Master’s-granting state university. The campus is located in a rural setting with a predominantly non-Hispanic white population (~75%, U.S. Census Bureau, 2021). The service area is home to nine Native American Tribes and resides on Wiyot ancestral land. The majority of first-year students are full-time and residential, and most students come from large urban centers elsewhere in California (San Francisco Bay Area and Southern California), with only 6% of students from the local area. The institution was federally designated a Hispanic Serving Institution after exceeding 25% Latinx undergraduate enrollment in starting in 2013.

At this institution, first-year STEM learning communities are designed to be cohorts of first-year undergraduate students pursuing similar majors and engaged in an interdisciplinary place-based theme. We therefore call them “place-based learning communities”. Each place-based learning community is composed of five interacting components: (1) a several-day “Summer Immersion” program with hands-on science, social justice, and welcoming activities immediately preceding the fall semester, (2) block enrollment in lower division courses that include some linked content, (3) a peer mentoring program, (4) a first-year experience course, and (5) an (optional) living learning opportunity in the campus residence hall. Additional details on the general design and structure of place-based learning communities, as well as the centrality of Indigenous perspectives to place-based education, are available in Seawright (2014), Johnson et al. (2017), Sprowles et al. (2019), Johnson et al. (2020), and in short videos describing the program (Appendix A). It is important to note that nearly learning community participants experience all five components (~85% chose the optional living learning component) while students in the reference group experienced none of them. Thus, with this design we cannot isolate the impact of any single component. Rather, the analysis shows possible impact of the entire first-year learning community experience.

This study examines outcomes of the first place-based learning community developed for STEM students, which began in fall 2015. Called the Klamath Connection, it links practices shown to improve first-year college student performance to a major feature of our geographic location: The Klamath River. The Klamath River Basin extends from Southern Oregon to the mouth of the river in northern California, an area encompassing over 15,750 miles^2^. It is inhabited by 120,000 people, 13% of which are Native American. Multiple environmental and social justice issues are associated with the region, such as conflicts over water rights and natural resource conservation, and issues affecting a diverse group of communities that include four Native American tribal nations: the Yurok, Hoopa, Karuk and Klamath Tribes. The issues of the Klamath are complex, engaging, and conspicuously multidisciplinary, providing a rich and nuanced context in which to explore interconnectedness of disciplines. The program involves students, faculty, and staff, many who have experience and expertise in the region, as well off-campus community partners including professional scientists, local Native American tribal members, and environmental restoration groups. Through integrated curriculum and activities, the program offers a substantively re-imagined first year experience for STEM students.

The first three cohorts of the Klamath Connection place-based learning community were 63, 116, and 118 students respectively, admitted in fall 2015, 2016, and 2017. The first cohort was composed of students enrolled in one the campus’s four largest STEM majors: Biology, Environmental Science, Wildlife, or Zoology. Students majoring in Botany, Environmental Resource Engineering, or Fisheries were added to the subsequent cohorts. First-year students admitted to one of the included majors were invited to “opt in” via paper and electronic invitations followed by more personalized calls and emails from staff and faculty. Overall, more than 95% lived on campus in their first year. To simplify block-scheduling of the first cohort, only students ready for college-level math were included in the program. For the second and third cohorts, all students admitted to their majors were invited to participate regardless of math preparedness. This opt-in approach and college-ready requirement for the first cohort necessitated choosing an appropriate reference groups of students to minimize self-selection bias in outcomes (described in the Data Analysis section, below).

## Methods

We used a quasi-experimental design to compare academic outcomes of three cohorts of learning community students relative to a reference group of students. We used propensity score matching to identify the reference group. Students were matched with the MatchIt package in R (Ho et al., 2018; R Core Team, 2018), using two continuous variables (high school GPA and number of AP units completed) and 4 binary variables: whether or not a student self-reported as female, from an underrepresented group, first-generation, and whether the student was designated by university admissions as being “college ready” in math, meaning their first math course would be college algebra or higher. We aimed to achieve a 2:1 match (2 reference for each learning community participant) and set the caliper width to 0.2. Limits in availability of matching students yielded an eventual match of 1.88:1.

Analyses involved multiple response variables, described in detail below, to assess students’ longer-term academic outcomes in STEM. For all analyses we used four binary indicators as grouping variables: learning community vs. reference, and yes/no status for underrepresented group, first-generation, and low-income. Underrepresented group (operationalized as African American, American Indian, Latinx, or two or more including one of those races/ethnicities) and first-generation status were assigned based on self-reported student admissions data; low-income status was defined by whether or not a student received a federal Pell grant. A student could belong to more than one student group. Cohort year (2015, 2016, 2017) was included as an additional covariate in initial models but since it was not significant it was removed from subsequent analyses. We tested global models with all possible interactions, and when they were non-significant we emphasized additive models and models with only the URG × Learning Community interaction because closing race-ethnicity equity gaps was a primary emphasis of our work.

To assess the program’s effect on academic achievement, we used ANOVA on grade-point-average (GPA) and units completed at the institution after students’ second, third, and fourth years, and compare them to effects after students’ first years. In addition, we identified four core STEM courses students in these majors typically take in or near their sophomore year (introductory biology, calculus, physics, and statistics) and four more they take in their junior or senior year (genetics, plant taxonomy, mammalogy, and organic chemistry) that also often have comparatively low success rates, large equity gaps, and large sample sizes. Not all students in the learning community were required to take each of these courses, but these courses nonetheless represented a range of different upper division subjects and had large enough numbers of students for analyses. A student grade of A, B, C, or Credit (a “passing” grade or mark) was categorized as a “success,” while a D, F, or unauthorized withdrawal was considered a “non-success.” Students receiving an incomplete or withdrawing early were removed from this analysis. For descriptive purposes (see Results) we also report the proportion of students earning each grade. We used general linear models with logit link (logistic regressions) to examine success in each course using the same grouping variables described above.

To examine retention, we quantified which students were still enrolled at the institution in the fall semester of their second, third, and fourth years (“institutional retention”), and which of these retained students were still in a declared STEM major (“STEM retention”). Likewise, we quantified which students graduated after four years, either from the institution regardless of major (institutional 4-year graduation) or from a STEM major specifically (STEM 4-year graduation). Both retention and graduation values are reported as the percentage of students retaining or graduating. We used general linear models with logit link to examine institutional and STEM retention and graduation using our grouping variables.

Lastly, we analyzed results from a “first destination survey” administered by the career advising center at the institution. All students scheduled to graduate with a STEM degree in spring of 2019, 2020, and 2021, corresponding to four years after first-year cohorts beginning in 2015–2017, were electronically invited to participate in the survey. Survey questions were a mixture of standard questions provided by the Handshake® First Destination Survey and approved by the National Association of Colleges and Employers (Handshake, 2021), as well as six additional questions we added specifically to assess students’ self-identified sense of STEM identity and STEM career preparedness (Table 2). Here, we report on results from these additional six questions. Students responded to affirmative statements on a Likert scale ranging from (1) strongly disagree, (2) slightly disagree, (3) neutral, (4) slightly agree, to (5) strongly agree. Numerical values were analyzed using an ANOVA with participation in the learning community vs. the matched reference as the grouping variable. The overall survey response rate was 26%, so it is important to note that data were available only for a subset of the learning community (*n* = 28, 27% response) and the reference group (*n* = 56, 31% response) with only a portion of those belonging to an underrepresented group (*n* = 7 and 16, respectively). Therefore, this analysis does not adhere to a true quasi experimental design as with the other response variables; we present results as preliminary descriptive findings, and we did not disaggregate data for this analysis by the other student groups.

All data were obtained from the institution’s Office of Institutional Effectiveness and all analyses were conducted in R (R Core Team, 2018) with α = 0.05. The effect sizes are reported as Hedges’ g (for continuous variables) or Cox’s d (for binary responses; What Works Clearinghouse, 2017). We interpret small effects as Hedges’ g and Cox’s d value of 0.2–0.49, and large effects as values >0.8, with medium effect sizes in between these values (Chen et al., 2010). All data collection, management, and analysis was completed under approval of the campus’s Institutional Review Board (IRB #15–238 and #17–200).

## Results

A total of 297 students initially participated in the learning community in 2015–16, 2016–17, and 2017–18. Some students (27) declined to self-report one or more demographic variable, they were removed from further analysis, yielding data for 270 students included in analyses, which corresponded to 18.1% of incoming first-time STEM students over these three years. After propensity score matching, the matched reference group was initially statistically similar to the participants, with all Hedges’ g and Cox’s d below thresholds for small effect sizes, except for the proportion of low-income students (Table 1; What Works Clearinghouse, 2017). The vast majority of students from an underrepresented group at this university are Latinx (~82% in our study population).

**Table 1:** Student demographics of students in the Klamath Connection learning community and a comparative reference group all first-year STEM students

Variable	Learning Community (n = 270)	Reference Group (n = 508)	Reference vs. Learning Hedge’s g or Cox’s d^a^
High School GPA	3.49 ± 0.41	3.51 ± 0.45	0.047
AP Units	10.97 ± 13.56	11.53 ± 12.71	0.047
% College-ready math	86%	85%	0.049
% First-generation	44%	46%	0.030
% Low-income	36%	44%	0.182
% Female	62%	62%	0.006
% White	54%	54%	0.048
% Underrepresented group (URG)^b^	39%	40%	0.017
% African American	1%	2%	
% American Indian	1%	1%	
% Asian American	4%	2%	
% Latinx	32%	33%	
% Two or more races	9%	7%	

^a^Students are from academic years 2015–16, 2016–17, and 2017–18, and the reference group was identified by propensity score matching (see Methods), Hedges’ g (for High School GPA and AP units) and Cox’s d values (other variables) are provided, demonstrating baseline equivalence between the learning community and reference group of students

^b^We operationalize students from underrepresented groups (URG) as those from the following groups: African American, American Indian, Latinx, and two or more including one of these races/ethnicities

**Fig. 1:**
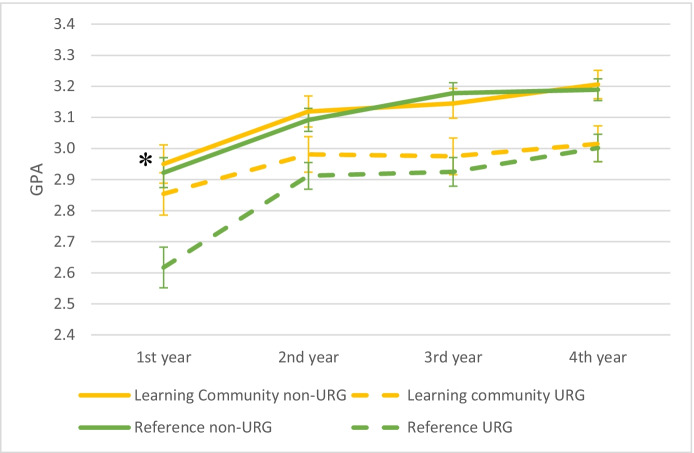
Grade point averages (GPA on 4-pt scale, ±1 SE) after students’ first, second, third, and fourth years for participants in a learning community and for a matched reference group; data are disaggregated by students in an underrepresented group (URG) or not (non-URG). Asterisks indicates a small Hedge’s g effect size of the learning community for URG student in the first year only

Learning community participants continued to show a higher number of completed units into the long term, but the benefits of GPA diminished over time. Specifically, the small but significant increase in units earned after the first year for learning community participants (+3.4 units) persisted into the second, third, and fourth years (+3.7, +3.9, +4.1 units; F_1,567_ = 4.63, *P* = 0.03, F_1,509_ = 4.32, *P* = 0.04, F_1,472_ = 3.85, *P* = 0.05, respectively). For underrepresented students, the effect was slightly larger (+7.5, +7.6, +8.3, and + 8.7 units after their first to fourth years, respectively). Likewise, the benefits of participating in a learning community on units earned also extended to first-generation and low-income students (Table 4 in Appendix B). In contrast, while previous work showed a small increase in the mean first year GPAs for underrepresented students in the learning community (+0.24 grade point units; Johnson et al., 2020), GPAs rose overall in the second and third years, and the impact of learning community participation diminished to near zero (Fig. 1, all F < 0.6, *P* > 0.05). Similarly, initially strong but then diminishing benefits of learning community participation on GPA were also observed for first generation and low-income students (Table 4 in Appendix B).

**Fig. 2:**
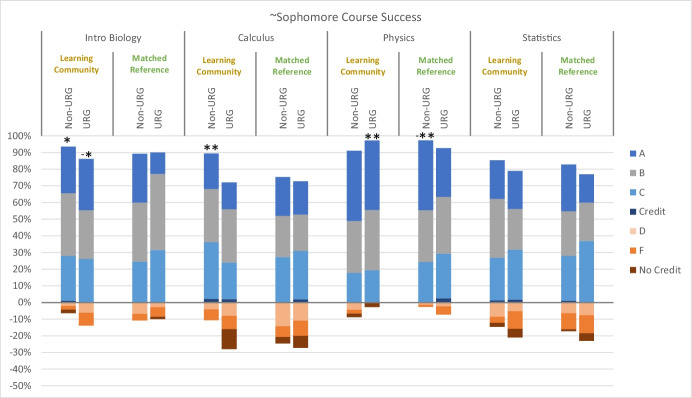
Grade distributions for selected core STEM courses typically taken in or near students’ Sophomore year for participants in a learning community and for a matched reference group; data are disaggregated by student in a underrepresented group (URG) or not (non-URG). Asterisks indicate small (*) or medium (**) Cox’s d effect sizes for higher pass rates for learning community participants than for the matched reference, or the reverse (−)

**Fig. 3:**
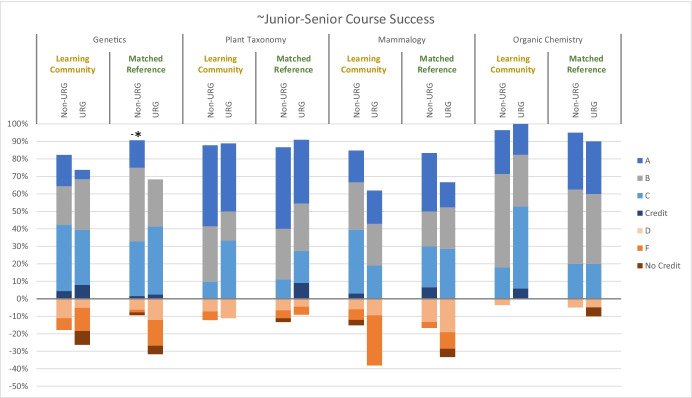
Grade distributions for selected core STEM courses typically taken in or near students’ junior or senior years for participants in a learning community and for a matched reference group; data are disaggregated by student in a underrepresented group (URG) or not (non-URG). Asterisks indicate small (*) or medium (**) Cox’s d effect sizes for higher pass rates for learning community participants than for the matched reference, or the reverse (−)

Previous work showed a significant benefit of the learning community on first year STEM course outcomes (Johnson et al., 2020), but the current study shows that effects on participants’ academic performance in sophomore and junior/senior level STEM courses were generally insignificant and inconsistent. None of the logistic regressions on success rate for sophomore courses was significant (all *P* > 0.05), though there were some noteworthy effect sizes (Table 5 in Appendix B). Among sophomore courses (Fig. 2), course success rates were higher for non-underrepresented learning community participants than for the matched reference in a core introductory biology course and in calculus, but these effect sizes did not extend to underrepresented students. In contrast, there was a benefit of learning community participation for underrepresented students in physics, but this effect was actually reversed for non-underrepresented students in physics (Fig. 2). Effects of participation in the learning community on grades in statistics were negligible. A similar pattern of varying to negligible effects was seen for first-generation and low-income students (Table 4 in Appendix B). None of the logistic regressions on success rate for junior/senior courses was significant (all *P* > 0.05), and there were even fewer noteworthy effect sizes of learning community participation on grades among junior/senior level courses (Fig. 3; Table 5 in Appendix B). For non-underrepresented students, the pass rate in genetics was lower for learning community participants than for the reference group, though proportionally more As were earned by learning community participants. For underrepresented students, the pass rate in organic chemistry was higher for learning community participants than for the reference group. Effect sizes of participation in the learning community on grades in plant taxonomy and mammalogy were negligible. Likewise, most effect sizes were negligible or small for first-generation and low-income students (Table 5 in Appendix B).

**Fig. 4:**
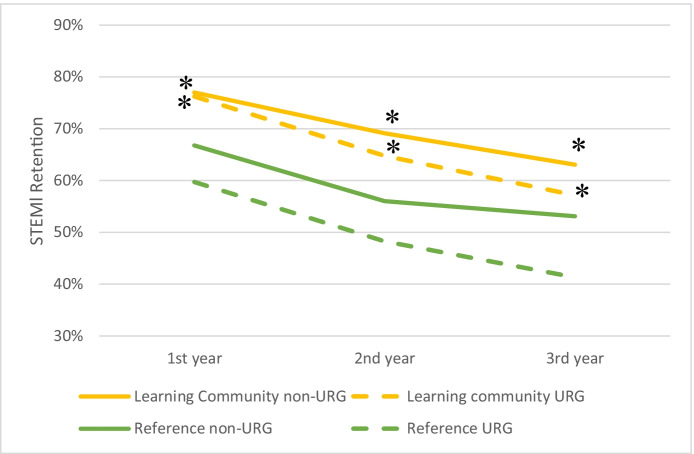
STEM retention after students’ first, second, and third years for participants in a learning community and for a matched reference group; data are disaggregated by students in an underrepresented group (URG) or not (non-URG). Asterisks indicate small Cox’s d effect size of the learning community for URG and non-URG in all three years

Despite the modest and diminishing effects of learning community participation on grades over time, there remained strong positive effects of the learning community on students’ retention into their second, third, and fourth years (Fig. 4). Specifically, first year retention in STEM was higher for learning community participants for both non-underrepresented and underrepresented students (+8% and + 17%; respectively; z = 2.65, *P* < 0.01), and this effect remained strong for second year retention (+13% and + 17%, respectively; z = 3.39, *P* < 0.01), and only diminished slightly for third year retention (+10% and + 16%; z = 1.94, *P* = 0.05). Moreover, equity gaps in STEM retention were consistently narrower for learning community participants than for the reference group (Fig. 4). Benefits of learning community participation on institutional retention (not necessarily in a STEM major) after students’ first, second, and third years were also observed for both underrepresented and non-underrepresented students, ranging from +6% to +12%. Likewise, benefits of participation in a learning community on retention extended to first-generation and low-income students (Table 4 in Appendix B).

**Fig. 5:**
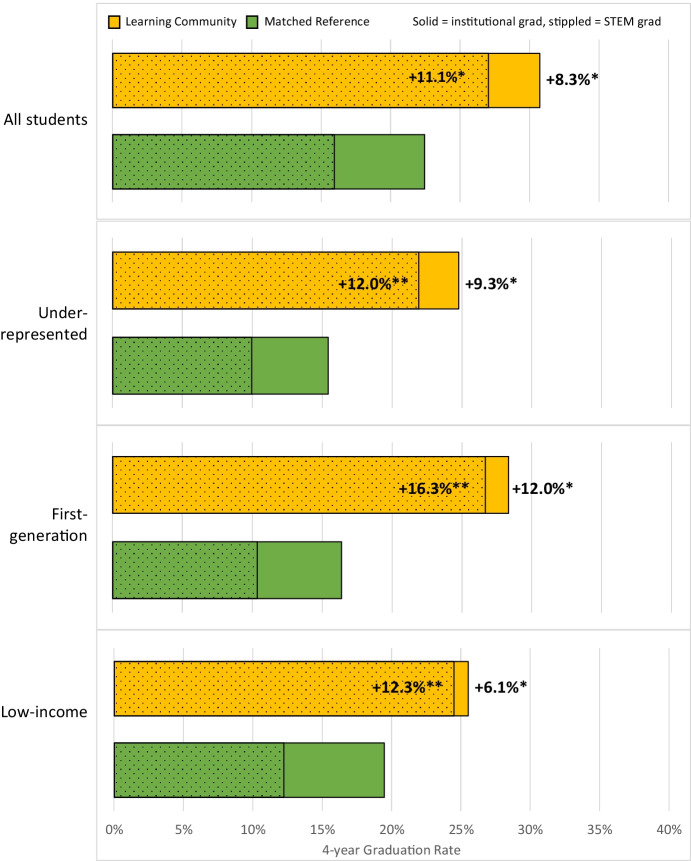
Four-year institutional graduation rates (solid) and STEM-specific graduation (stippled) for participants in a learning community and for a matched reference group; data are shown for all students, students from an underrepresented group, first-generation students, and low-income students; see text for definitions. Asterisks indicate small (*) or medium (**) Cox’s d effect sizes for higher rates for both institutional and STEM graduation for all student groups

**Fig. 6:**
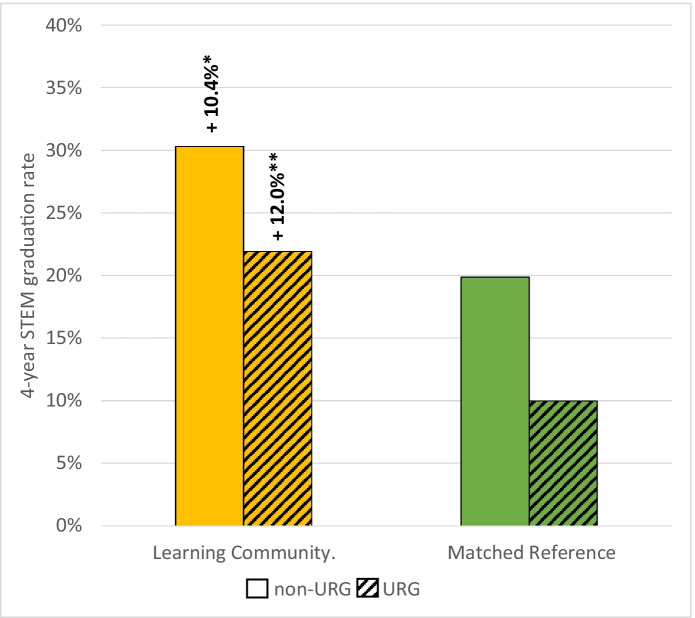
Four-year STEM-specific graduation rates for participants in a learning community and for a matched reference group; data are disaggregated by those in an underrepresented group (cross hatched) or not (solid) to illustrate equity gaps. Asterisks indicate substantial effect sizes of the learning community for both URG and non-URG student groups, though the difference between them (the equity gap) was only narrowed slightly in the learning community

Four-year graduation rates were significantly improved by participation in the learning community. Among all students, graduation rates were marginally higher for participants than the matched reference both from the institution (+8.3%, z = 1.72, *P* = 0.08), and significantly higher in STEM specifically (+11.1%, z = 2.68, *P* < 0.01; Fig. 5). Benefits of participation were higher for students from underrepresented groups (+9.3% and + 12.0% for institutional and STEM graduation, respectively) and for first-generation students (+12.0% and + 16.3%). The benefits for low-income students were also strong (+6.1% and + 12.3%, for institutional and STEM graduation, respectively; Fig. 5). However, equity gaps in graduation between underrepresented and non-underrepresented students were only slightly narrowed in the learning community, and a substantial gap (−8.4%) persisted (Fig. 6). Among students that had not yet graduated but were still enrolled in STEM (i.e., continuing) versus those that had left the university, we again found a strong positive effect of the learning community participation (+ 4.6% overall, +7.5% for URG, z = 3.167, *P* < 0.01; Table 4 in Appendix B).

**Fig. 7:**
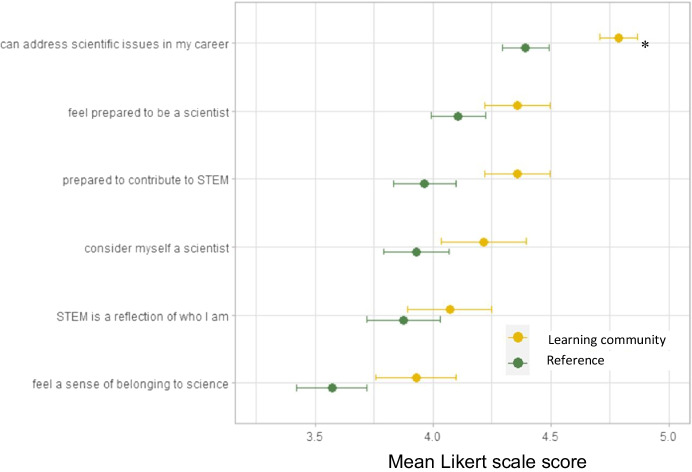
Mean (±1 SE) responses to questions about STEM career preparedness and identity self-reported by graduates who started in the learning community (gold) and by those in a matched reference group (green). The sample size (28 and 56, respectively) is a non-random subset of the larger pools of students included in the other analyses. Responses were on a 5-point Likert scale (from 1-strongly disagree to 5-strongly agree). Asterisk indicates a significant difference in means between the two samples. See Table 2 for the full question prompts

Students’ responses to a graduation survey revealed that, on average, learning community participants consistently reported higher scores on questions about STEM career preparedness and STEM identity than did students in the reference group, though this difference was significant for only the question about being prepared to address scientific issues, and effect sizes for all questions were below 0.4 (F_1,91_ = 8.56, *P* < 0.05; Fig. 7). Generally, a benefit of learning community participation was more apparent on questions about sense of belonging and career preparation (e.g., can address scientific issues, prepared to contribute to STEM) and less strong for sense of STEM identity (e.g., consider myself a scientist, STEM is a reflection of who I am).

**Table 2:** Questions used to assess graduating students’ self-reported sense of STEM career preparedness and STEM identity. Responses were on a 5-point Likert scale ranging from (1) strongly disagree, (2) slightly disagree, (3) neutral, (4) slightly agree, to (5) strongly agree

To what extent do you agree with the following statements:	
1. My educational experiences at HSU have helped me understand how my career can help address scientific environmental social and civic issues faced by our society.	
2. I am prepared to be a scientist, an engineer, or a mathematician.	
3. I consider myself a scientist, an engineer, or a mathematician.	
4. I have a strong sense of belonging to the community of scientists, engineers, or mathematicians.	
5. I feel prepared to actively contribute to the STEM community.	
6. Being a scientist an engineer or a mathematician is an important reflection of who I am.	

## Discussion

Increasing the number of students entering the STEM workforce and advancing inclusive excellence for undergraduate STEM students is urgently needed both to address social inequity and to improve the quality of science (Gibbs, 2014; Asai, 2020). A growing body of evidence suggests first-year learning communities can improve academic outcomes and narrow equity gaps for students in their first critical year (Weiss et al., 2015). However, far less evidence is available to assess whether these early benefits persist into students’ later years. Results of this study suggest that a first-year place-based learning community continued to improve some outcomes for students into their second, third, and fourth years. Specifically, we observed significant increases in the number of units students completed and year-to-year retention at the institution and in STEM in particular, especially for students from underrepresented groups and for first-generation students. These benefits to low-income students were also significant, but smaller in magnitude. In contrast, increases in students’ GPAs and outcomes in core STEM courses that were initially strong for learning community participants gradually diminished in later years to the point that they were statistically indistinguishable with those of students in the matched reference group (Figs. 1, 2, 3). Nonetheless, 4-year graduation rates in STEM were 11 percentage points higher for all learning community participants combined, and impacts were slightly higher when data were disaggregated for underrepresented, first-generation, and low income students (12–16%, Fig. 5), which narrowed but did not erase equity gaps (Fig. 6; Table 4 in Appendix B). These results support other studies that show learning communities may be a useful practice to increase the number of students earning STEM degrees, especially when operating in concert with other high-impact practices (Kuh, 2008; Maltby et al., 2016; Weiss et al., 2015), but it is important to note that equity gaps still persisted in graduation (Fig. 6). Moreover, a survey of graduating seniors, though based on a limited sample, suggested that the learning community also enhanced students’ sense of STEM identity and STEM career preparedness (Fig. 7), a result with hopeful implications for how learning communities practiced at scale could contribute to diversifying the future STEM workforce. Our results are generally consistent with Dagley et al. (2016) and Cambridge-Williams et al. (2013), who found persistent benefits to first-year retention and long-term graduation from first-year learning communities at two large universities with very active research (R1 in Carnegie Classification of universities) in Florida and Virginia, respectively. In contrast, Whalen and Shelley (2010) found no significant effect of learning community participation on graduation of STEM students at a midwestern R1 university, although there were benefits for non-STEM students.

There are several possible explanations for persistent benefits of the learning community on retention and graduation despite diminishing effects on grade acquisition. First, our learning community may have its strongest impacts on psycho-social factors, such as fostering a sense of belonging and community, and they may be less effective at improving and narrowing gaps in academic skills. This interpretation is consistent with results reported in a previous analysis of first-year outcomes of this same learning community (Johnson et al., 2020). Specifically, we previously found that the learning community increased 6 of 10 students’ self-reported score on psycho-social factors, whereas the same survey revealed no significant differences in students’ self-reported scores on academic skills. Comments and other survey information from our learning community participants suggest that the Summer Immersion component of our learning community was especially impactful (Johnson et al., unpubl.), particularly the hands-on activities in the field and laboratory with faculty, peers, and community partners.

Second, the academic effects of our learning community may occur especially in the first year because students’ use of academic supports may diminish afterward. Our first-year learning community curriculum actively promotes the use of academic support services such as the tutoring center and supplemental instruction, and we know that learning community participants used these services more than did the reference group (Johnson et al., upubl. data), but whether that difference diminishes over time is unknown. Moreover, the first-year learning community includes class sessions that focus on study strategies (e.g., exam wrapper and retention practices; Brown et al., 2014) with the hope that participating students may adopt these habits for longer-term benefit, but we have not quantified whether this is true, and we are aware of no studies that have examined this important question specifically. Research into these and other possible explanations for diminishing effects on academic outcomes is needed to inform how to best design learning community curricula for lasting benefits.

A third reason the benefits of the learning community on grades may decline after the first year is that the intentional weaving of place-based curriculum in the curricular and co-curricular activities of our first-year learning community occur primarily in the first year. The Klamath River theme and the associated content that link and span several courses all reside in the first year curriculum, with relatively little explicit return to these details later. Moreover, while many courses taken later by the students in these majors touch on science and social justice, none does so in a way that spans multiple classes and connects to a cohort’s shared field experience. Thus, the nexus of community, STEM content, and social justice advanced by the place-based learning community in this study is likely more conspicuous to students in their first year than in later years. Future work should emphasize scaffolding lessons from first-year learning communities into support in later years to sustain and possibly magnify early benefits. Other researchers have suggested that enhancing the relevance of science to social justice issues can promote inclusive excellence (Chamany et al., 2008; Sheardy, 2010; Hewitt et al., 2019). Our group is examining these students’ perceptions of the learning community curricula as it relates to science and social justice, whether the recognition of those links is stronger for some students than others, and whether that recognition is associated with improved long-term learning outcomes (Sprowles et al., in prep).

It is important to acknowledge that with our learning community design, it is impossible to disentangle effects of its individual components, many of which are high impact practices in and of themselves. It is well documented that student participation in one or more of the original ten High Impact Practices (HIPs) are linked to improved student outcomes and increased entry into the workforce after graduation, particularly for traditionally underserved populations including underrepresented and first generation students (Kuh & O'Donnell, 2013; Wawrzynski & Baldwin, 2014). Therefore, as our campus is working to institutionalize five place-based learning communities to service all incoming first-year STEM students (~400 students), most of the components are being successfully scaled up and sustained. Several commentators have warned that an erosion in the fidelity of learning communities to their core tenets as they expand and scale up, including the strength of the linked content, could result in smaller benefits to students (Mintz, 2019; Lederman, 2020). For example, Sanchez et al. (2006) found that peer mentoring coupled with an orientation course, a partial learning community as we define it here (Smith et al., 2009), yielded first-year benefits but no effects on longer term retention and graduation. Indeed, we have found it is increasingly difficult to maintain linked content for five different learning communities across courses, especially in large core STEM courses composed of a range of students from several different learning communities. The possibility of eroded effects should be watched vigilantly and guarded against by administrators and key faculty and staff involved in learning community coordination.

Results of this study suggest the potential for even stronger benefits of first-year learning communities if their impacts can be scaffolded in the students’ second and later years. The “sophomore slump” is a phenomenon that is widely suggested because of the particular social (e.g., moving off campus) and academic (e.g., finalizing major choices) demands students face (Milsom et al., 2014), but it has received only piecemeal research (Yorke, 2015). At the institution in this study, grades and year-to-year retention rise from year one to year two overall, so a true sophomore slump may not be widespread among these students. Nonetheless, the academic benefits of the first-year learning community in this study clearly declined after year one, so actions taken to extend these benefits into later years would likely help advance inclusive excellence at the institution, as it has at others (Dagley et al., 2016). Indeed, there is no silver bullet to raising academic performance and closing equity gaps, and multi-year integrated support will certainly outperform any intervention in a single year. Future work should examine how first-year learning communities can connect with and propel students toward these other powerful high impact practices. In particular, we believe that future research should examine the impact of more sustained connections between social justice, civic engagement, and STEM classroom activities, as well extra- and co-curricular experiential learning opportunities such as paid internships and research assistant positions on university projects which are well-described HIPs for inclusive student success in STEM (Graham et al., 2013; Hernandez et al., 2018; Jensen et al., 2021). Qualitative data from student interviews or focus groups also enrich the understanding of students’ experiences in the learning communities (Dean & Dailey, 2020).

This study is among the first to provide evidence for longer-term benefits of a first year learning community for STEM students in promoting inclusive student success for a Master’s-granting institution in a remote rural setting unlike that of the familial homes of many of its students. These results are in alignment with others who have hypothesized that campuses with largely residential and full-time student populations may benefit most from fostering a sense of belonging with first-year learning communities, as they assist students far away from their familial homes connect to a new community (Weiss et al., 2015). Critics have noted that unless campuses can balance student integration with maintaining connections to families and communities back home, learning communities may be inappropriate for students of some cultures and/or for campuses with large commuter populations (González, 2002). As the expanding list of Hispanic-Serving Institutions is increasingly including campuses in settings like ours, our place-based model may be encouraging to other campuses challenged with welcoming students to localities without large local Hispanic populations (sensu HACU, 2021).

Lastly, our results suggested lasting benefits on retention and graduation and should arm administrators and student advocates with evidence of the potential for learning communities to be a wise investment in the pursuit of inclusive student success. Indeed, the economic benefits of increased retention alone may at least partially offset the costs of implementing and operating learning communities. However, the effects of the learning community on academic outcomes declined as students progressed into later years, and while several equity gaps were narrowed, few were erased. Additional work is needed to better understand how to leverage high-impact practices in first-year learning communities to advance durable improvements in students’ grade acquisition and engagement on other curricular, co-curricular, and extra-curricular activities demonstrated to improve entry into the STEM workforce (Estrada, 2014; Hernandez et al., 2018).

## Appendix A

**Table 3:** Short videos and links describing the Klamath Connection place-based learning community program and providing full descriptive and analytical statistics.

Video title	URL
Place-based learning communities at Humboldt State University	https://www.youtube.com/watch?v=aOKzgH5Ztk0&list=PL8xMAoml8kSEHr0y5MhiHulil2gDFW34u&index=4
Klamath Connection at Humboldt State University	https://www.youtube.com/watch?v=oJV7Flv5qnM&list=PL8xMAoml8kSEHr0y5MhiHulil2gDFW34u&index=3
Traditional Ecological Knowledge & Place-based Learning Communities	https://www.youtube.com/watch?v=liKV74avPso&t=80s

## Appendix B

**Table 4:** Full descriptive statistics of all response variables for the first three cohorts of the Klamath Connection place-based learning community (LC) compared to a reference group (REF) disaggregated by underrepresented groups (URG), first-generation, and low-income students.

	Group	All students				URG		Non-URG				First generation			Low Income	
Response variable		mean	SE	n	mean	SE	n	mean	SE	n	mean	SE	n	mean	SE	n
Units earned after 1 year	LC	40.2	0.996	254	39.6	1.708	99	40.5	1.218	155	40.2	1.548	112	40.3	1.700	91
	REF	36.8	0.770	470	32.1	1.123	186	39.9	1.001	284	33.2	0.992	213	33.9	1.050	201
Units earned after 2 years	LC	70.6	1.300	213	70.2	2.252	81	70.9	1.585	132	71.9	2.110	90	68.8	2.243	68
	REF	66.9	0.979	359	62.6	1.312	136	69.5	1.330	223	64.4	1.250	156	64.3	1.336	150
Units earned after 3 years	LC	99.5	1.475	197	97.3	1.664	120	100.8	1.706	124	99.8	2.332	81	98.3	2.663	63
	REF	95.6	1.158	317	89.0	1.664	73	99.6	1.496	197	90.9	1.628	134	93.0	1.600	132
Units earned after 4 years	LC	125.3	1.664	173	121.6	2.966	65	127.6	1.958	108	126.0	2.516	72	122.9	3.214	48
	REF	121.2	1.255	305	114.9	1.918	116	125.1	1.587	189	117.4	1.862	130	119.4	1.961	123
GPA after 1 year	LC	2.912	0.046	254	2.854	0.068	99	2.95	0.062	155	2.94	0.074	112	2.882	0.085	91
	REF	2.801	0.039	470	2.617	0.065	186	2.922	0.048	284	2.658	0.061	213	2.679	0.060	201
GPA after 2 years	LC	3.067	0.038	213	2.981	0.057	81	3.119	0.050	132	3.116	0.059	90	3.01	0.061	68
	REF	3.024	0.029	359	2.912	0.043	136	3.092	0.037	223	2.955	0.039	156	2.926	0.043	150
GPA after 3 years	LC	3.082	0.038	197	2.975	0.059	73	3.145	0.048	124	3.109	0.057	81	3.042	0.060	63
	REF	3.082	0.028	317	2.925	0.046	120	3.178	0.034	197	2.946	0.044	134	3.008	0.045	132
GPA after 4 years	LC	3.134	0.036	173	3.015	0.058	65	3.206	0.046	108	3.122	0.060	72	3.043	0.073	48
	REF	3.118	0.036	173	3.002	0.044	116	3.189	0.035	189	3.009	0.043	130	3.05	0.044	123
Success rate in intro biology	LC	90.5%	143	158	86.2%	56	65	93.5%	87	93	95.6%	65	68	87.5%	49	56
	REF	89.5%	179	200	90.0%	63	70	89.2%	116	130	88.2%	75	85	92.7%	76	82
Success rate in calculus	LC	80.4%	78	97	72.0%	36	50	89.4%	42	47	74.5%	41	55	77.1%	27	35
	REF	74.2%	98	132	72.7%	40	55	75.3%	58	77	76.7%	46	60	74.6%	44	59
Success rate in physics	LC	93.8%	76	81	97.2%	35	36	91.1%	41	45	93.0%	40	43	93.5%	29	31
	REF	95.7%	110	115	92.7%	38	41	97.3%	72	74	94.2%	49	52	95.3%	41	43
Success rate in statistics	LC	82.7%	115	139	78.9%	45	57	85.4%	70	82	86.4%	51	59	78.4%	40	51
	REF	80.4%	127	158	76.9%	50	65	82.8%	77	93	76.8%	53	69	79.7%	51	64
Success rate in genetics	LC	78.3%	65	83	73.7%	28	38	82.2%	37	45	76.2%	32	42	68.6%	24	35
	REF	81.9%	86	105	68.3%	28	41	90.6%	58	64	68.5%	37	54	72.1%	31	43
Success rate in plant taxonomy	LC	88.1%	52	59	88.9%	16	18	87.8%	36	41	90.0%	27	30	85.0%	17	20
	REF	88.1%	59	67	90.9%	20	22	86.7%	39	45	83.3%	20	24	84.0%	21	25
Success rate in mammalogy	LC	75.9%	41	54	61.9%	13	21	84.8%	28	33	71.4%	20	28	59.1%	13	22
	REF	76.5%	39	51	66.7%	14	21	83.3%	25	30	68.2%	15	22	84.2%	16	19
Success rate in organic chemistry	LC	97.8%	44	45	100.0%	17	17	96.4%	27	28	100.0%	21	21	100.0%	16	16
	REF	93.3%	56	60	90.0%	18	20	95.0%	38	40	86.2%	25	29	84.6%	22	26
Institutional retention after 1 year^A^	LC	81.5%	220	270	81.0%	85	105	81.8%	135	165	76.7%	92	120	74.5%	73	98
	REF	72.6%	369	508	70.1%	141	201	74.3%	228	307	69.4%	161	232	71.0%	157	221
Institutional retention after 2 years	LC	75.9%	205	270	70.5%	74	105	79.4%	131	165	67.5%	81	120	66.3%	65	98
	REF	63.6%	323	508	60.2%	121	201	65.8%	202	307	58.2%	135	232	59.7%	132	221
Institutional retention after 3 years	LC	69.6%	188	270	63.8%	67	105	73.3%	121	165	65.0%	78	120	63.3%	62	98
	REF	62.2%	316	508	58.2%	117	201	64.8%	199	307	56.9%	132	232	57.5%	127	221
STEM retention after 1 year^A^	LC	76.7%	207	270	76.2%	80	105	77.0%	127	165	72.5%	87	120	67.3%	66	98
	REF	64.0%	325	508	59.7%	120	201	66.8%	205	307	59.1%	137	232	58.8%	130	221
STEM retention after 2 years	LC	67.4%	182	270	64.8%	68	105	69.1%	114	165	63.3%	76	120	60.2%	59	98
	REF	53.0%	269	508	48.3%	97	201	56.0%	172	307	47.0%	109	232	48.0%	106	221
STEM retention after 3 years	LC	60.7%	164	270	57.1%	60	105	63.0%	104	165	60.0%	72	120	48.0%	47	98
	REF	48.4%	246	508	41.3%	83	201	53.1%	163	307	40.5%	94	232	38.5%	85	221
Institutional 4-year graduation	LC	30.7%	83	270	24.8%	26	105	34.5%	57	165	28.3%	34	120	25.5%	25	98
	REF	22.4%	114	508	15.4%	31	201	27.0%	83	307	16.4%	38	232	19.5%	43	221
STEM 4-year graduation	LC	27.0%	73	270	21.9%	23	105	30.3%	50	165	26.7%	32	120	24.5%	24	98
	REF	15.9%	81	508	10.0%	20	201	19.9%	61	307	10.3%	24	232	12.2%	27	221
Institutional “continuation”^A^	LC	21.9%	59	270	27.6%	29	105	18.2%	30	165	22.5%	27	120	23.5%	23	98
	REF	15.9%	81	508	16.9%	34	201	15.3%	47	307	15.1%	35	232	14.0%	31	221
STEM “continuation”^A^	LC	18.1%	49	270	21.0%	22	105	16.4%	27	165	18.3%	22	120	18.4%	18	98
	REF	13.6%	69	508	13.4%	27	201	13.7%	42	307	12.1%	28	232	10.9%	24	221

^A^Continuation described the percentage of students that had not graduated in 4 years but were still enrolled into their fifth year (institutional or STEM-specific enrollment)

**Table 5:** Analytical statistics of all response variables for the first three cohorts of the Klamath Connection place-based learning community compared to a reference group disaggregated by underrepresented groups (URG), first-generation (FG), and low-income (LI) students. Hedges’s g effect sizes and ANOVA statistics of the main effect of learning community participation (*F*, df, and *P*) are shown for continuous response variables (the first eight), and Cox’s d effect sizes and logistic regression statistics of the main effect of learning community participation (*z*, *P*) are shown for the remaining 18 binary variables. Bolded values indicate a small or medium (if also italicized) effect size, and a significant (*P *< 0.05) main effect of learning community participation.

	Hedge’s g or Cox’s d effect sizes					Test statistics and *P*		
Response variable	All	URG	Non-URG	FG	LI	*F* or *z*	df	*P*
Units earned after 1 year	**0.207**	**0.470**	0.037	**0.461**	**0.42**	**6.832**	**1,718**	**0.009**
Units earned after 2 years	0.198	**0.437**	0.073	**0.431**	**0.26**	4.652	1,566	**0.031**
Units earned after 3 years	0.189	**0.491**	0.059	**0.451**	**0.27**	4.357	1,507	**0.037**
Units earned after 4 years	0.187	**0.305**	0.117	**0.403**	0.16	3.849	1,472	**0.050**
GPA after 1 year	0.137	**0.288**	0.035	**0.329**	**0.24**	2.761	1,717	**0.097**
GPA after 2 years	0.078	0.136	0.048	**0.312**	0.16	0.486	1,565	0.493
GPA after 3 years	0.000	0.099	-0.066	**0.318**	0.07	0.004	1,507	0.949
GPA after 4 years	0.034	0.027	0.035	0.226	-0.01	0.055	1,471	0.815
Success rate in intro biology	0.068	**-0.222**	**0.338**	***0.640***	**-0.357**	0.809		0.130
Success rate in calculus	**0.214**	-0.022	***0.610***	-0.069	0.084	0.942		0.346
Success rate in physics	**-0.223**	***0.610***	***-0.756***	-0.122	**-0.208**	-1.789		0.074
Success rate in statistics	0.095	0.071	0.116	**0.394**	-0.046	0.172		0.863
Success rate in genetics	-0.136	0.157	**-0.444**	**0.232**	-0.101	-1.764		0.078
Success rate in plant taxonomy	0.004	-0.133	0.061	**0.351**	0.045	0.024		0.981
Success rate in mammalogy	-0.018	-0.123	0.068	0.092	***-0.776***	0.208		0.835
Success rate in organic chemistry	***0.689***	na	0.211	na	na	0.205		0.837
Institutional retention after 1 year^A^	**0.306**	**0.358**	**0.269**	**0.224**	0.105	1.869		0.062
Institutional retention after 2 years	**0.358**	**0.276**	**0.420**	**0.242**	0.172	**3.100**		**0.002**
Institutional retention after 3 years	**0.201**	0.143	**0.242**	**0.206**	0.147	1.888		0.059
STEM retention after 1 year	**0.373**	**0.466**	**0.308**	**0.365**	0.222	**2.433**		**0.015**
STEM retention after 2 years	**0.368**	**0.410**	**0.340**	**0.404**	**0.300**	**3.330**		**0.001**
STEM retention after 3 years	**0.302**	**0.387**	**0.248**	**0.477**	**0.235**	**2.703**		**0.007**
Institutional 4-year graduation	**0.259**	**0.357**	**0.214**	**0.425**	**0.211**	1.724		0.085
STEM 4-year graduation	**0.405**	**0.563**	**0.340**	**0.694**	**0.512**	2.684		**0.007**
Institutional “continuation”^A^	0.235	**0.380**	0.125	**0.297**	**0.382**	2.648		**0.008**
STEM “continuation”^A^	0.208	**0.324**	0.127	**0.298**	**0.371**	3.167		**0.002**

^A^Continuation described the percentage of students that had not graduated in 4 years but were still enrolled into their fifth year (institutional or STEM-specific enrollment).
